# Motor lumbosacral radiculopathy in HIV-infected patients

**DOI:** 10.4102/sajhivmed.v20i1.992

**Published:** 2019-10-28

**Authors:** Kaminie Moodley, Pierre L.A. Bill, Vinod B. Patel

**Affiliations:** 1 Department of Neurology, University of KwaZulu-Natal, Durban, South Africa

**Keywords:** HIV, lumbosacral radiculopathy, ART, corticosteroids, treatment outcome

## Abstract

**Background:**

This study is a review of the clinical findings and treatment outcome of 11 HIV-infected patients with motor lumbosacral radiculopathy.

**Objectives:**

To describe the clinical, laboratory, electrophysiological features and treatment outcome in HIV-infected motor lumbosacral radiculopathy which is a rare manifestation of HIV.

**Method:**

A retrospective review of HIV-infected patients with motor lumbosacral radiculopathy was performed at Inkosi Albert Luthuli Central Hospital (IALCH), Durban, South Africa between 2010 and 2015.

**Results:**

Eleven black African patients met the inclusion criteria. There were six women. The median age was 29 years, the interquartile range (IQR) was 23–41 years, the median duration of symptom progression was 6.5 months (IQR 3–7.5 months). The median CD4 count was 327 cells/µL (IQR 146–457). The cerebrospinal fluid (CSF) median polymorphocyte count was 0 cells/µL (IQR 0 cells/µL – 2 cells/µL), lymphocyte count was 16 cells/µL (IQR 1 cells/µL – 18 cells/µL), glucose level was 3.1 mmol/L (IQR 2.8 mmol/L – 3.4 mmol/L) and protein level was 1.02 g/dL (IQR 0.98 g/dL – 3.4 g/dL). All patients were treated with corticosteroid therapy. Ninety-one per cent recovered fully within 6 months of treatment, the median time for recovery was 3.4 months (IQR 1.8–5.6 months). There were no relapses during the 18-month follow-up.

**Conclusion:**

HIV-infected patients with motor lumbosacral radiculopathy responded to corticosteroids, with no relapses during the 18-month follow-up period.

## Introduction

Progressive lumbosacral polyradiculopathy is a well-described complication of late HIV infection and is usually caused by opportunistic infections such as cytomegalovirus (CMV), herpes simplex virus (HSV), varicella zoster virus (VZV), Ebstein-Barr virus (EBV), syphilis, tuberculosis (TB), cryptococcus and, less commonly, lymphoma, paraneoplastic polyradiculopathy, chronic inflammatory demyelinating polyradiculopathy (CIDP), or diffuse infiltrative lymphocytosis (DILS).^1,2,3^ Infective aetiologies, lymphoma and paraneoplastic polyradiculopathy are usually subacute and are progressive unless treated.^1,3,4,5,6,7^

In 2000, Benatar et al. described four HIV-infected patients who presented with acute or subacute weakness with spontaneous recovery.^8^ Infective and inflammatory aetiologies were excluded. This entity was described as a ‘unique’ clinical entity in the setting of HIV or a ‘variant of Guillain–Barre syndrome (GBS)’.^8^ Since 2000, there were no further documented cases in the literature.

Between 2010 and 2015, we retrospectively identified a similar cohort of 11 HIV-infected patients who presented with a motor lumbosacral radiculopathy. In this article, we add to the current literature regarding this unusual group of patients by describing the clinical presentation, demographic features, electrodiagnostic, radiological, cerebrospinal fluid (CSF) findings and response to therapy.

## Methods

Patients were identified between 2010 and 2015 in the Department of Neurology at Inkosi Albert Luthuli Central Hospital, which is a 1000-bed tertiary hospital in Durban, KwaZulu-Natal province, South Africa. The Department of Neurology attends approximately 8000 patients per year.

The inclusion criteria for patient selection were as follows: HIV-infected patients older than 18 years with lower motor neuron weakness involving exclusively the lower limbs, normal sensation, preserved sensory nerve action potentials (SNAPs) and lumbosacral root enhancement on magnetic resonance imaging (MRI). Exclusion criteria were as follows: abnormal sensation on clinical examination, upper limb or truncal involvement, upper motor neuron signs, sensory nerve action potential on nerve conduction studies that were less than 70% of normal values, compressive or intra-spinal lesions accounting for the weakness, polyradiculopathies due to infective, malignant or paraneoplastic aetiology, clinical features of DILS or raised creatinine kinase levels with electrophysiological or histological features of a myopathy, and electrolyte abnormalities, for example hypokalaemia accounting for weakness and areflexia.

Data extracted from patient records included clinical findings, laboratory results, electrodiagnostic findings (nerve conduction and needle electromyography), MRI of the thoracolumbar and lumbosacral spine, duration of therapy and response to therapy.

Tests that were conducted to exclude infective or neoplastic causes of a polyradiculopathy included CSF polymerase chain reaction (PCR) for VZV, CMV, HSV, EBV; CSF Ziehl–Neelson (ZN) stain, culture and gene expert for TB; CSF Venereal Disease Research Laboratory (VDRL), fluorescent treponemal antibody absorption (FTA-ABS) for syphilis; CSF cytology for malignancy (lymphoma); CSF cryptoccocal antigen, India ink stain and cryptococcal culture; chest radiograph for pulmonary tuberculosis (TB), CSF cytology, paraneoplastic antibodies and MRI spine for structural and inflammatory and/or infective lesions.

Patients were followed up and scored according to the Modified Rankin Scale (mRS) to assess for relapses and response to therapy at 3-month intervals for 6 months and thereafter 6 monthly up to 18 months.

### Ethical considerations

This article followed all ethical standards for research without direct contact with human or animal subjects.

## Results

### Clinical features, cerebrospinal fluid, electrophysiological and magnetic resonance imaging findings

Eleven patients met the inclusion criteria. There were six women. The median age was 29 years (interquartile range [IQR] 23–41 years). All patients were of black African ancestry. The mean duration of symptom progression (continuous and not stepwise) was 6.5 months (IQR 3–7.5 months). No patients had preceding flu-like illness, sensory complaints or upper limb symptoms. Examination revealed that they had flaccid, symmetrical areflexic paraparesis with normal assessment of mental state, cranial nerves and upper limbs. Sensory testing for all modalities was normal and sphincters were normal.

CD4 counts are listed in Table 1 (median CD4 count of 327 cells/µL, IQR 146–457). None of the patients were on antiretroviral therapy (ART) at the time of diagnosis. However, all patients were referred to ART clinics for monitoring or initiation of ARTs according to the South African ART guidelines applicable during the study period. Blood investigations, which included routine tests such as full blood count, urea and electrolytes, autoimmune screen (anti-nuclear factor, anti-neutrophil cytoplasmic antibodies), paraneoplastic antibodies, creatinine kinase, rapid plasma reagin test, vitamin B12 and folate, glucose and serum protein electrophoresis, did not reveal any abnormalities.

**TABLE 1:** Demographic, laboratory, and radiological features of motor lumbosacral radiculopathy.

Number	Age (Years)	CD4 count at diagnosis (cells/μL)	mRS Scores at presentation	Duration of progression of symptoms (Months)	CSF				MRI (RE)	Time to recovery (Months)	mRS at 18 months	Relapses within 18 months
					P	L	Glu	Prot				
1.	27	657	4	3.0	0	32	3.2	0.98	Y	3	0	Nil
2.	22	155	4	2.0	0	0	3.7	1.02	Y	2	0	Nil
3.	42	480	5	4.0	0	16	2.3	3.68	Y	3	0	Nil
4.	29	149	5	3.5	0	4	3.1	1.83	Y	4	0	Nil
5.	21	265	5	2.0	2	17	3.3	1.02	Y	5	0	Nil
6.	24	450	4	4.0	1	18	2.8	1.69	Y	4	0	Nil
7.	18	380	4	4.0	0	24	3.6	2.61	Y	3	0	Nil
8.	51	140	4	2.0	0	0	3.4	0.77	y	2	0	Nil
9.	32	124	4	5.0	1	12	4.2	1.52	Y	3	0	Nil
10.	40	112	5	4.0	2	9	4.1	0.89	Y	5	0	Nil
11.	43	389	4	2.0	0	5	3.2	1.34	Y	4	1	Nil

mRS, Modified Rankin Scale; CSF, cerebrospinal fluid; MRI, magnetic resonance imaging; RE, root enhancement; P, polymorphocyte count (cells/μL); L, lymphocyte count (cells/μL); Glu, glucose (mmol/L); Prot, protein (g/dL); Y, yes.

The CSF median polymorphocyte count and lymphocyte count were 0 cells/µL (IQR 0–2) and 16 cells/µL (IQR 1 cells/µL – 18 cells/µL), respectively. The CSF median glucose and protein was 3.1 mmol/L (IQR 2.8 mmol/L – 3.4 mmol/L) and 1.02 g/dL (IQR 0.98 g/dL – 3.4 g/dL), respectively (Table 1). The CSF tested negative for viruses (CMV, HSV, HTLV1, EBV and VZV), TB, syphilis and cryptococcus. CSF cytology was negative. Five patients had negative antiganglioside antibodies, which were not tested for in the other six patients.

Motor and sensory electrophysiological tests are listed in Tables 2 and 3, respectively. Normal values for IALCH electrophysiology laboratory are listed in Table 4. The compound muscle action potential (CMAP) of the tibial and peroneal nerves were reduced in amplitude, with median CMAP of 3.6 mV (IQR 2.2–4.2) and 3.5 mV (IQR 2.6–4.2), respectively. The distal motor latency (DML) and conduction velocity (CV) were within the normal range for both the tibial and peroneal nerves. The *F* responses were either absent or prolonged, with median 62 ms (IQR 59–70.5) and 68 ms (IQR 64–70) for the peroneal and tibial nerves, respectively, compared to the respective *F* estimates of 53 ms (IQR 50–55) and 54 ms (IQR 52–55). There were no conduction blocks or temporal dispersion. The sural and superficial peroneal SNAPs were present in all patients, although amplitudes were marginally reduced, most likely because of coexistent HIV peripheral neuropathy. The median sural and superficial peroneal SNAP was 12.5 µV (IQR 10–13) and 6.5 µV (IQR 5.7–7.1), respectively, which is greater than 80% the expected lower limit of normal (Table 4). The peak sensory latencies for both nerves were normal: median 4.1 ms (IQR 3.9–4.2) and 3.1 ms (IQR 2.27–3.3) for the sural and superficial peroneal, respectively. The upper limb motor and sensory nerve conduction tests were performed in 7 of the 11 patients (63%) and were normal (Tables 2 and 3).

**TABLE 2:** Electrophysiological findings of patients with motor lumbosacral radiculopathy in HIV-infected patients: Motor studies.

Motor nerve	Pat: 1	Pat: 2	Pat: 3	Pat: 4	Pat: 5	Pat: 6	Pat: 7	Pat: 8	Pat: 9	Pat: 10	Pat: 11	Median	IQR
**Peroneal ( R/L)**													
Amplitude (mV)	2.2/2.4	4.2/3.8	1.9/2.3	4.4/3.8	2.6/1.8	1.8/2.6	4.1/4.4	5.2/4.8	0.9/1.1	3.4/3.6	5.2/5.1	3.6	2.2–4.3
DML (ms)	5.2/4.8	4.8/5.1	6.2/6.1	5.9/5.8	5.4/5.6	6.3/6.2	5.8/5.9	5.5/4.9	6.9/7.1	4.6/4.2	4.4/4.2	5.5	4.8–6.5
CV (m/s)	44/46	46/48	41/43	40/41	44/46	41/43	44/45	42/43	38/41	42/44	38/40	43	41–44
F Response (ms)	58/61	62/68	59/58	73/75	78/68	59/58	59.5/61	abs/64	abs/abs	62/67	73/75	62	59–70.5
F Estimate (ms)	51/50	49/50	52/53	58/54	51/53	52/53	54/55	48/49	abs/abs	57/58	61/62	53	50–55
**Tibial ( R/L)**													
Amplitude (mV)	3.1/3.5	2.8/2.6	1.8/2.1	4.2/4.4	3.2/3.8	2.8/2.6	3.9/4.1	6.7/7.2	1.2/2.1	2.8/2.6	6.1/6.8	3.5	2.6–4.2
DML (ms)	7/6.6	5.8/5.6	6.8/6.6	4.8/4.9	5.9/6.1	5.8/5.6	4.3/4.5	5.3/5.5	6.8/6.9	6.8/6.6	5.9/6.1	3.5	2.6–4.2
CV (m/s)	48/49	40/41	42/44	44/45	51/48	41/40	45/46	43/42	40/39	41/40	43/44	45	43–46
F Response (ms)	62/65	70/68	64/74	64/62	65/68	69/70	abs/70	65/72	abs/71	69/70	64/62	68	64–70
F Estimate (ms)	52/52	54/56	48/49	54/55	51/52	54/56	55/abs	53/54	58/abs	54/56	56/54	54	52–55
**Median ( R/L)**													
Amplitude (mV)	8.2/8.5	10.2/10	n/a	n/a	n/a	12/12.8	8.6/8.1	10.9/11.8	n/a	11.1/10.9	6.1/6.2	10.3	8.2–11
DML (ms)	3.2/3.3	4.1/4.2	n/a	n/a	n/a	4.1/3.8	3.9/3.8	4.4/4.5	n/a	4.1/4.2	3.9/3.8	4	3.8–4.2
CV (m/s)	58/52	58/56	n/a	n/a	n/a	55/56	48/45	44/45	n/a	51/50	49/51	51	48–56
F Response (ms)	28/29	31/29	n/a	n/a	n/a	24/28	24/26	28/32	n/a	31/33	29/28	29	27.5–31
F Estimate (ms)	31/30	30/31	n/a	n/a	n/a	28/28	32/30	33/34	n/a	35/32	32/34	31	30–31
**Ulnar ( R/L)**													
Amplitude (mV)	6.9/6.3	5.8/5.2	n/a	n/a	n/a	8.8/9.1	7.9/6.1	9.8/9.1	n/a	8.6/9.1	9.4/9.2	8.8	6.6–9.1
DML (ms)	2.6/2.1	3.3/3.1	n/a	n/a	n/a	2.9/2.6	2.6/2.1	3.1/2.7	n/a	2.9/2.6	3.2/2.8	2.7	2.6–3
CV (m/s)	50/48	58/56	n/a	n/a	n/a	54/56	52/49	46/48	n/a	52/54	52/56	52	49–55
F Response (ms)	31/29	26/24	n/a	n/a	n/a	28/25.5	31/29	31/30	n/a	28/25.5	31/30	29	26–30
F Estimate (ms)	30/30	26/27	n/a	n/a	n/a	30/31	33/32	34/35	n/a	30/31	34/35	31	30–33

R/L, Right/Left; DML, distal motor latency; CV, conduction velocity; IQR, interquartile range.

**TABLE 3:** Electrophysiological findings in patients with motor lumbosacral radiculopathy in HIV-infected patients: Sensory studies.

Sensory nerves	Pat:1	Pat:2	Pat:3	Pat:4	Pat:5	Pat:6	Pat:7	Pat:8	Pat:9	Pat: 10	Pat:11	Median	IQR
**Sural**													
Amplitude (µV) R/L	9.8/10.5	12.5/12.3	11.2/12.3	10.3/9.5	11.5/11.6	12.2/12.9	14.1/13.9	9.5/9.3	16/15.8	12.2/13.1	15.1/15	12.5	10–13
Peak latency (ms) R/L	4.1/4.3	3.8/4	4.2/4.1	3.9/4.1	4.2/3.8	4.4/4.2	3.9/4.1	3.9/3.8	4.4/4.5	2.9/3.1	3.9/3.6	4.1	3.9–4.2
**Superficial Peroneal**													
Amplitude (µV) R/L	3.5/4	6.7/6	5.6/5	4.8/5	6.6/6	7.3/7.5	6.5/6.1	7.1/7.4	6.8/6.5	7.1/7.7	7.8/7	6.5	5.7–7.1
Peak latency (ms) R/L	3.1/3.3	2.9/2.8	2.6/2.8	3.2/3.3	3.8/3.6	2.1/2.5	2.2/2.6	3.2/3.8	3.1/2.9	3.5/3.3	3.2/3	3.1	2.27–3.3
**Median**													
Amplitude µV R/L	45/58	68/62	n/a	n/a	n/a	85/76	58/56	115/98	n/a	45/48	85/96	65	56.5–85
Peak latency R/L	3.4/3.6	3.3/3.1	n/a	n/a	n/a	2.6/2.9	2.7/2.9	3.1/2.9	n/a	3.1/3.3	2.9/3.2	3	2.9–3
**Ulnar**													
Amplitude µV R/L	24/28	45/44	n/a	n/a	n/a	48/53	77/76	65/64	n/a	32/38	38/27	44	33–61
Peak latency R/L	2.2/2	2.1/2.6	n/a	n/a	n/a	2.8/3	2.1/2.7	2.8/2.6	n/a	2.6/2.3	2.1/2.3	2	2.3–2.8

R/L, Right/Left; IQR, interquartile range.

**TABLE 4:** Normal values for Inkosi Albert Luthuli Central Hospital Electrophysiology Laboratory.

Nerve	Distal motor latency (ms)	Amplitude	NCV (m/s)	*F* latency (ms)
Peroneal nerve (EDB)	< 6.5	> 4mV	> 41	< 57
Tibial nerve ( AHB)	< 5.9	> 4mV	> 40	< 57
Ulnar nerve (ADM)	< 3.6	> 6mV	> 51	< 32
Median nerve (APB)	< 4.5	> 4mV	> 48	< 33
Sural (Stimulation site = 14cm)	< 4.5	> 7uV	-	-
Superficial peroneal (Stimulation site = 14cm)	< 3.8	> 7uV	-	-
Ulnar SNAP	< 2.1	> 10uV	-	-
Median SNAP	< 2.3	> 15uV	-	-

Source: In-house collaboration among neurophysiologists from Pretoria Academic and Groote Schuur Hospital, SA Neurology Association Meeting, Rustenburg, 1998

EBD, Extensor Digitorium Brevis; AHB, Abductor Hullucis Brevis; ADM, Adductor Digiti Minimi; APB, Abductor Pollicis Brevis; SNAP, Sensory Nerve Action Potential; ms, millisecond; NCV, nerve conduction velocity; m/s, metre per second.

Needle electromyography (EMG) findings are listed in Table 5. Muscles examined included the lumbar paraspinals (lower and mid lumbar), gluteus medius, quadriceps, tibialis anterior and gastrocnemius. These muscles demonstrated neurogenic changes as evidenced by increased insertional activity, positive sharp waves, fibrillation potentials, and reduced or single unit recruitment of polyphasic motor unit potentials with greater involvement of proximal rather than distal muscles.

**TABLE 5:** Needle Examination.

Muscle	Spontaneous activity				Motor unit potential			Recruitment pattern
	Insertional activity	Fib	PSW	Fasic	Amplitude	Duration	Polyphasia	
Lumbar paraspinals	2+ (3+ in patient 11, 7)	3+	2+	0	normal	normal	3+	Reduced (Single unit recruitment in patient 11)
Gluteus medius	1+	2+	1+	0	normal	normal	2+	Reduced (Single unit in patient 1,9,11)
Quadriceps	1+	1+	1+	0	normal	normal	2+	Reduced (Single unit recruitment in patient 11)
Tibialis anterior	1+	1+	1+	0	normal	normal	1+	Reduced
Gastrocnemius	1+	1+	1+	0	normal	normal	1+	Reduced

Fib, Fibrillation potentials; PSW, Positive sharp waves; Fasic, Fasiculations.

**FIGURE 1:**
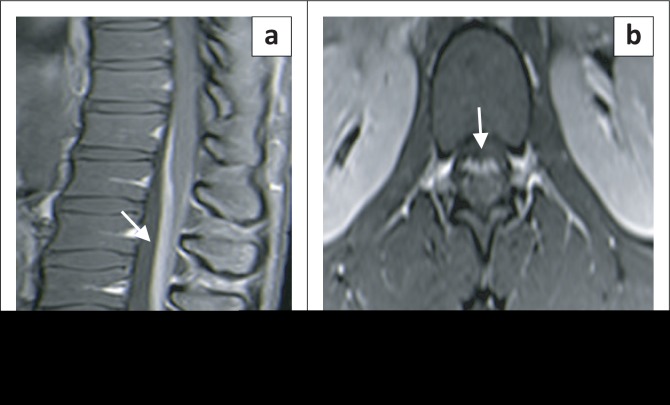
(a) Post-gadolinium sagittal and (b) axial lumbosacral spine images showing ventral root enhancement (arrows).

All 11 patients had MRI with gadolinium, of the thoracolumbar and lumbosacral spine. Imaging revealed root enhancement of the lumbosacral ventral roots in all patients (Figure 1). There were no identifiable structural abnormalities and no thoracic root enhancement.

All patients were treated with corticosteroids (prednisone) at an initial dose of 1.5 mg/kg/day at diagnosis for 4–6 weeks or longer if needed. Thereafter corticosteroid therapy was tapered and stopped based on side effects or response to therapy. This was done at the discretion of the attending neurologist. Sixty-four per cent (7/11) of patients showed a clinical response within the first 4 weeks of treatment and recovered fully by 3 months. In this category, corticosteroids were given at full dose for 4 weeks, then tapered over the subsequent 6–8 weeks and stopped by 3 months. Thirty-six per cent (4/11) of patients received initial full-dose corticosteroids for longer periods of 4–6 weeks as they had taken longer to respond, and then corticosteroids were tapered over the subsequent 18 weeks. Eighteen per cent (2/11) of patients recovered fully by 4 months and the other 18% (2/11) by 5 months. In this category of ‘slower responders’, corticosteroid therapy was stopped by 6 months. All patients had no residual clinical deficit except patient 11, who despite demonstrating a good response to corticosteroid therapy by 4 months had minimal residual deficit at 18 months follow-up with a mRS of 1. The median time for recovery in all categories was 3.4 months (IQR 1.8–5.6).

There were no relapses during the 18-month follow-up. Within the period of corticosteroid therapy, there were no documented side effects and no patients required corticosteroid sparing immunosuppressive agents or long-term corticosteroids therapy. Six patients had CD4 counts < 350 cells/µL and qualified for ART according to ART guidelines at that time. HIV titres were not documented. Three patients were commenced on ART at 4 months after the diagnosis. These three patients had recovered prior to ART commencement. The other three patients were commenced on ART 6 months after presentation. At 18 months follow-up, seven patients were on ART.

## Discussion

The 11 patients presented in this article represent an unusual cohort of HIV-infected patients with a subacute motor lumbosacral radiculopathy. Sensory, sphincter function and upper limbs were normal in all patients.

The MRI showed gadolinium enhancement confined to the lumbar ventral roots. In other infective or inflammatory aetiologies, such as syphilis, TB, viral infections or lymphoma, both dorsal and ventral roots are involved, enhancement may be nodular and patchy with coexistent myelitis, intramedullary granulomas, subdural collections or discitis especially in infective aetiologies.^9,10,11^

The clinical scenario of symmetrical ascending weakness, areflexia and high CSF protein is suggestive of GBS.^12,13^ More recently, the boundaries of GBS have expanded and variations include a paraparetic GBS where the upper limbs and cranial nerves are spared.^14^ A further variant is associated with HIV seroconversion.^15,16^ These patients typically have a CSF pleocytosis as seen in our cohort, which may reflect HIV viral replication in the CSF space rather than immunological changes that occur with the subacute motor lumbosacral radiculopathy.^17,18^ The axonal variant of GBS, associated with a high rate of preceding *Campylobacter jejuni* infection, may present as a pure motor axonopathy.^19^ Our patients may meet some of the criteria for a ‘variant GBS’.^19^ Benatar et al. described four patients with similar clinical findings. They described these patients as a possible variant of GBS or a distinct clinical entity.^8^ However, the unusual features include duration of progression, limitation of signs to the lower limbs, CSF pleocytosis and response to corticosteroid therapy, which is known not to be of benefit in GBS.^20,21^

The above cohort may therefore be consistent with a proximal motor variant of CIDP involving demyelination of the ventral roots rather than GBS. Evidence for the above includes prolonged or absent *F* responses with normal DMLs, neurogenic changes in the paraspinals, ventral root gadolinium enhancement on MRI, raised CSF protein and rapid response to corticosteroid therapy with no relapses. Denervation on needle EMG may suggest secondary axonal loss. Moodley et al. described CIDP in the setting of HIV. In that particular cohort of patients, demyelination was distal rather than proximal, patients had sensory and motor symptoms rather than exclusively motor manifestations, and both upper and lower limbs were involved.^22,23^

The rapid response to corticosteroid therapy and the predilection for ventral roots may suggest an antibody-mediated process that targets the ventral roots only. The production of these antibodies may be a transient phenomenon during the course of HIV infection as none of the patients relapsed during the 18-month follow-up despite stopping corticosteroid therapy for 6 months or less. We hypothesise that immune reconstitution with ART may have prevented relapses by induction of tolerance, by increasing the number of functional T regulatory cells and hence maintaining remission. Some diseases associated with HIV may recover with immune reconstitution, for example HIV-associated CIDP, HIV-associated motor neuron syndrome or even myasthenia gravis, despite there being insufficient literature to support the above.^22,24^ Therefore, variable or unexpected patterns can occur in HIV immune reconstitution, with exacerbation of some diseases and improvement of others.

The wide range of CD4 counts may also support an immune-mediated process, which is independent of the stage of HIV. The high CD4 counts in some patients were not explained by concomitant DILS as the patients had no clinical features of DILS.^1,25^

Seven of the 11 patients were on ARTs at 18-month follow-up. However, only three patients started ART at 4 months after presentation. These three patients had recovered prior to commencing ART on corticosteroid therapy alone. By 6 months all patients had recovered. Hence, the recovery was likely induced by corticosteroid therapy as no patients showed spontaneous recovery before corticosteroid therapy. However, it is likely that ART-induced immune reconstitution may have prevented relapses as all patients on ART at 18 months follow-up had CD4 counts above 350 cells/µL.

Since the South African government’s rollout programme in 2017 which commenced all HIV-infected patients on ART at the time of diagnosis irrespective of CD4 counts, we rarely encounter the above group of patients. This further supports an immune basis for the disease which may improve with changes in tolerance.

Limitations of this study include retrospective design, small patient numbers and lack of a control arm.

## Conclusion

Studies are required to understand the pathogenesis of this disease in order to identify the possible antigenic targets. This may help refine therapy in HIV-uninfected patients with pure immune-mediated motor polyradiculopathy, for example paraneoplastic and other immune ventral root radiculopathies.^5,6,7,26,27,28^

## References

[1] Centner CM, Bateman KJ, Heckmann JM Manifestations of HIV infection in the peripheral nervous system. Lancet Neurol. 2013;12:295–309. 10.1016/S1474-4422(13)70002-4 23415569

[2] Ferrari S, Vento S, Monaco S, et al Human immunodeficiency virus-associated peripheral neuropathies. Mayo Clin Proc. 2006;81:213–219. 10.4065/81.2.213 16471077

[3] Miller RF, Fox JD, Thomas P, et al Acute lumbosacral polyradiculopathy due to cytomegalovirus in advanced HIV disease: CSF findings in 17 patients. J Neurol Neurosurg Psychiatry. 1996;61:456–460. 10.1136/jnnp.61.5.456 8937337 PMC1074040

[4] Ingber S, Buckstein R Paraneoplastic lumbosacral axonal polyradiculopathy preceding the diagnosis of nodular lymphocyte predominant Hodgkin lymphoma: A case report. Leuk Lymphoma. 2008;49:2009–2011. 10.1080/10428190802276289 18720211

[5] Murphy SM, Khan U, Alifrangis C, et al Anti Ma2-associated myeloradiculopathy: Expanding the phenotype of anti-Ma2 associated paraneoplastic syndromes. J Neurol Neurosurg Psychiatry. 2012;83:232–233. 10.1136/jnnp.2010.223271 21205983 PMC3719382

[6] Rees J Paraneoplastic syndromes. Curr Opin Neurol. 1998;11:633–637. 9870129 10.1097/00019052-199812000-00004

[7] Rees JH Paraneoplastic syndromes: When to suspect, how to confirm, and how to manage. J Neurol Neurosurg Psychiatry. 2004;75 Suppl 2:ii43–50. 10.1136/jnnp.2004.040378 15146039 PMC1765657

[8] Benatar MG, Eastman RW Human immunodeficiency virus-associated pure motor lumbosacral polyradiculopathy. Arch Neurol. 2000;57:1034–1039. 10.1001/archneur.57.7.1034 10891986

[9] Brisset M, Chadenat ML, Cordoliani Y, Kamga-Tallom R, D’Anglejean J, Pico F [MRI features of neurosyphilis]. Rev Neurol (Paris). 2011;167:337–342. 10.1016/j.neurol.2010.08.012 21440277

[10] Marais S, Roos I, Mitha A, Mabusha SJ, Patel V, Bhigjee AI Spinal tuberculosis: Clinicoradiological findings in 274 patients. Clin Infect Dis. 2018;67:89–98. 10.1093/cid/ciy020 29340585

[11] Lipkin WI, Parry G, Abrams D, Kiprov D Polyradiculoneuropathy, polyradiculitis, and CMV in AIDS and ARC. Neurology. 1987;37:888 10.1212/wnl.37.5.888 3033548

[12] Shepherd SJ, Black H, Thomson EC, Gunson RN HIV positive patient with GBS-like syndrome. JMM Case Rep. 2017;4:e005107 10.1099/jmmcr.0.005107 29026634 PMC5610709

[13] Przedborski S, Liesnard C, Voordecker P, et al Inflammatory demyelinating polyradiculoneuropathy associated with human immunodeficiency virus infection. J Neurol. 1988;235:359–361. 10.1007/bf00314233 3171617

[14] >Van Den Berg B, Fokke C, Drenthen J, Van Doorn PA, Jacobs BC Paraparetic Guillain-Barre syndrome. Neurology. 2014;82:1984–1989. 10.1212/WNL.0000000000000481 24808021

[15] Pontali E, Feasi M, Crisalli MP, Cassola G Guillain-Barre syndrome with fatal outcome during HIV-1-Seroconversion: A case report. Case Rep Infect Dis. 2011;2011:972096 10.1155/2011/972096 22567484 PMC3336224

[16] Brannagan TH 3rd, Zhou Y HIV-associated Guillain-Barre syndrome. J Neurol Sci. 2003;208:39–42. 10.1016/s0022-510x(02)00418-5 12639723

[17] Ropper AH The Guillain-Barre syndrome. N Engl J Med. 1992;326:1130–1136. 1552914 10.1056/NEJM199204233261706

[18] Abdulle S, Hagberg L, Svennerholm B, Fuchs D, Gisslen M Cerebrospinal fluid viral load and intrathecal immune activation in individuals infected with different HIV-1 genetic subtypes. PLoS One. 2008;3:e1971 10.1371/journal.pone.0001971 18414666 PMC2291576

[19] Zhang HL, Wu J, Ni FM, et al Axonal variant of Guillain-Barre syndrome associated with campylobacter infection in Bangladesh. Neurology. 2010;75:194–195. 10.1212/WNL.0b013e3181cff735 20625176

[20] Hughes RA, Brassington R, Gunn AA, Van Doorn PA Corticosteroids for Guillain-Barre syndrome. Cochrane Database Syst Rev. 2016;10:CD001446 10.1002/14651858.CD001446.pub5 27775812 PMC6464149

[21] Wang YZ, Lv H, Shi QG, et al Action mechanism of corticosteroids to aggravate Guillain-Barre syndrome. Sci Rep. 2015;5:13931. 10.1038/srep13931 PMC4565078 26355080

[22] Moodley K, Bill PL, Patel VB A comparative study of CIDP in a cohort of HIV-infected and HIV-uninfected patients. Neurol Neuroimmunol Neuroinflamm. 2017;4:e315 10.1212/NXI.0000000000000315 28054000 PMC5182055

[23] Mochan A, Anderson D, Modi G CIDP in a HIV endemic population: A prospective case series from Johannesburg, South Africa. J Neurol Sci. 2016;363:39–42. 10.1016/j.jns.2015.11.013 27000218

[24] Moodley K, Bill PLA, Bhigjee AI, Patel VB A comparative study of motor neuron disease in HIV-infected and HIV-uninfected patients. J Neurol Sci. 2019;397:96–102. 10.1016/j.jns.2018.12.030 30597421

[25] Harrison TB, Smith B Neuromuscular manifestations of HIV/AIDS. J Clin Neuromuscul Dis 2011;13:68–84. 10.1097/CND.0b013e318221256f 22361691

[26] Anderson SC, Baquis GD, Jackson A, Monteleone P, Kirkwood JR Ventral polyradiculopathy with pediatric acute lymphocytic leukemia. Muscle Nerve. 2002;25:106–110. 10.1002/mus.1219 11754193

[27] Stefurak TL, Midroni G, Bilbao JM Vasculitic polyradiculopathy in systemic lupus erythematosus. J Neurol Neurosurg Psychiatry. 1999;66:658–661. 10.1136/jnnp.66.5.658 10209182 PMC1736344

[28] Ahn S-W, Yoon B-N Motor dominant polyradiculopathy with Primary Sjögren’s syndrome mimicking motor neuron disease. Ann Clin Neurophysiol. 2019;21:61–65. 10.14253/acn.2019.21.1.61

